# Construction and internal validation of a prediction nomogram for acquired premature ejaculation (APE) in PE patients

**DOI:** 10.1111/andr.12956

**Published:** 2020-12-26

**Authors:** Lei Zhang, Xinlong Dun, Guangdong Hou, Yu Zheng, Dongen Ju, Ping Meng, Fei Liu, Jiarui Yuan, Long Jin, Tao Jiang, Ming Gao, Jianlin Yuan

**Affiliations:** ^1^ Department of Urology Xijing Hospital Fourth Military Medical University Xi’an China; ^2^ St. George’s University School of Medicine West Indies Grenada; ^3^ Department of Nuclear Medicine Tangdu Hospital Fourth Military Medical University Xi’an China; ^4^ Department of Preventive Medicine Fourth Military Medical University Xi’an China; ^5^ Assisted Reproduction Center Northwest Women’s and Children’s Hospital Xi’an China; ^6^ Department of Andrology Xi’an Daxing Hospital Shaanxi University of Chinese Medicine Xi’an China

**Keywords:** nomogram, premature ejaculation, EPQ‐RSC

## Abstract

**Background:**

A predictive model for acquired premature ejaculation (APE) in PE patients has not yet been established.

**Objectives:**

This study was aimed at determining which factors were independently associated with the possibility of predicting APE in PE patients, and whether an effective pre‐treatment nomogram for predicting their individual chances of being APE in PE patients can be developed.

**Materials and methods:**

We analyzed the medical histories of 915 PE patients diagnosed at Xijing Hospital (Xi'an, China) and Northwest Women's and Children's Hospital (Xi'an, China) between May 2019 and May 2020. The diagnostic nomogram was developed using a multivariate logistic regression model by integrating selected significant variables determined through univariate analysis. Receiver operating characteristic curves were used to measure the predictive accuracy of the nomogram and its constituted variables, and calibrations were performed by making a comparison of nomogram‐predicted probability with actual rate of APE.

**Results:**

The independent predictors for APE that were identified include Age, Intra‐vaginal Ejaculation Latency Time (IELT), Frequency of sexual desire (FSD), and Eysenck Personality Questionnaire‐Revised Short Scale for Chinese (psychoticism) [EPQ‐RSC(P)] scores. The predictive accuracy of the nomogram was 0.782 (95% CI: 0.723–0.841). Also, excellent agreement was demonstrated between the nomogram‐predicted probability and the actual rate of APE.

**Discussion and conclusion:**

We identified 4 independent predictors for APE and demonstrated the potential significant differences in psychoticism between LPE and APE patients. This was the first internally validated predictive APE nomogram where good discrimination and calibration were applied, and it offers a promising role in clinical practice. More studies are necessary for verification of its universal applicability.

## 1 INTRODUCTION

Premature ejaculation (PE) is characterized as the most frequent sexual complaint, demonstrating a prevalence rate of 20–30%.^1^, ^2^, ^3^ In 2014, the International Society for Sexual Medicine (ISSM) defines PE and divides it into acquired PE (APE) and lifelong PE (LPE).^4^ Variable and subjective PEs have also been proposed for the classification of PE syndromes, but they have always been considered provisional.^5^ A Turkish study found that 20% of subjects complained of PE, and the rate of LPE was 2.3%, APE was 3.9%, variable PE was 8.5%, and subjective PE was 5.1%.^6^ Approximately 5% of prevalence of APE and LPE among the general population is consistent with epidemiological data, thus indicating that in nearly 5% of the population ejaculation latency is less than 2 minutes.^7^

It is believed that the pathogenesis of LPE is mainly caused by neurobiological and genetic issues,^8^ whereas APE is usually associated with sexual performance anxiety,^9^ psychological or relationship problems,^9^ erectile dysfunction (ED),^2^ prostatitis,^10^ and thyroid diseases,^11^ among others. It is imperative to determine the subtypes of PE and discuss patient expectations in detail prior to treatment. Although treatment of the underlying causes, such as ED, is the primary goal for APE, drug therapy is considered the first‐line treatment for LPE.^7^ Diagnosis and classification of PE are mainly based on the medical and sexual histories of patients,^7^ and intra‐vaginal ejaculation latency time (IELT) plays an important role in the classification of PE. Nomograms, graphical representation of multivariable prediction models, has been applied in the field of andrology and shows a good accuracy.^12^ However, nomogram prediction for APE in PE patients has not yet been established to date and it may be useful to establish a nomogram to predict APE in PE patients.

The predictors of PE subtypes are currently controversial, and the nomogram that predicts APE in PE patients has not yet been established. As such, this study aimed at determining which factors were independently associated with the possibility of predicting APE in PE patients, and whether an effective pre‐treatment nomogram can be developed to predict their individual chance of being APE in PE patients.

## 2 MATERIALS AND METHODS

### 2.1 Sample size assessment

Referring to previous studies,^13^ the P (Proportion) of APE in PE (including APE and APE) is at 60%; if the allowable error is 10% of P, it is at 0.06% and the Confidence Interval Width (Two‐Sided) is 0.12 and Alpha is 0.05. After the calculation of PASS software, version 11.0.7 (https://www.ncss.com/software/pass/), the minimum sample size was 271 which was a sufficient sample size for this study.

### 2.2 Patient population

We conducted an observational cross‐sectional study between May 2019 and May 2020 at Xijing Hospital (Xi'an, China) and Northwest Women's and Children's Hospital (Xi'an, China). A total of 915 PE patients (327 from Xijing Hospital and 588 from Northwest Women's and Children's Hospital) were included based on the diagnostic criteria for ISSM. The inclusion criteria involved a stable sexual relationship of at least 6 months and at least one sexual attempt within the last month. All participants were heterosexual and practiced vaginal sexual intercourse. A total of 252 LPE and 663 APE patients were identified through evaluation of the sexual and medical histories of patients by 2 experienced andrologists. All procedures performed in studies were in accordance with the 1964 Helsinki Declaration and its later amendments, or comparable ethical standards, and with the ethical standards of the institutional and national research committee. The Xijing Hospital Ethics Committee approved the study (KY20192108‐F‐1) and it was registered in Clinical Trials (https://clinicaltrials.gov/, NCT04235192). All participants provided written informed consent.

### 2.3 Study covariates

The 5‐item International Index of Erectile Function (IIEF‐5)^14^, ^15^ was used to evaluate erectile function, which has been widely used around the world including China.^16^ A patient was considered to have ED when the total IIEF‐5 score was below 22. Ejaculation function was assessed with the Premature Ejaculation Diagnostic Tool (PEDT),^17^, ^18^ which was linguistically validated in China in a previous study.^19^ Total of PEDT >10 indicates PE. IELT is the duration between vaginal penetration to ejaculation, and this was self‐reported in our study. History of masturbation was also assessed and frequency of sexual desire (FSD) was reported as “> 50% of the time” or “≤ 50% of the time”. We used the Patient Health Questionnaire‐9 (PHQ‐9)^20^ to assess the symptoms of depression, while the Generalized Anxiety Disorder (GAD‐7)^21^ was used to evaluate symptoms of anxiety, and both of them were validated in the Chinese population.^22^, ^23^ A total PHQ‐9 or GAD‐7 score ≥5 indicates the existence of depression or anxiety symptoms, respectively.

In our study, the Eysenck Personality Questionnaire‐Revised Short Scale for Chinese (EPQ‐RSC) version was used to evaluate patients’ personality status, which was translated and revised by Qian et al. in 2000^24^ and has since demonstrated good psychometric properties. This questionnaire includes 4 aspects: extraversion (E), neuroticism (N), psychoticism (P), and a lie (L) detector inventory. The raw scores of each subscale were added together and converted into T‐scores according to Chinese norms and were then classified as low (<40), normal (40–60), or high (> 60) when the questionnaire was completed.

### 2.4 Statistical analysis

R for Windows, version 3.6.1 (http://www.r‐project.org/) with the “rms” package was used for all statistical analyses. Categorical data were presented as frequencies (proportions) and were compared between subgroups by the Chi‐squared test. Continuous variables were presented as medians (ranges) and were compared by the Mann–Whitney U test. We performed a univariate logistic regression model to identify statistically significant variables, which were then incorporated in the multivariate model. The nomogram was developed using this final multivariate logistic regression model by integrating selected significant variables.

We measured the discriminatory capability of the nomogram according to the area under the receiver operating characteristic curve (AUC). The values ranged from a maximum of 1.0, which indicated a perfect discrimination, to a minimum of 0.5, which indicated a random chance. We generated calibration plots to test the consistency of prediction between the nomogram‐predicted probability and the actual outcome. In these calculations, bootstrapping with 1,000 resamples was performed.

Statistical significance was considered as *P* < 0.05.

## 3 RESULTS

### 3.1 Characteristics of variables

Statistically significant differences were demonstrated in PEDT (*P* < 0.001), IELT (*P* < 0.001), FSD (*P* = 0.039), and EPQ‐RSC(P) (*P* < 0.001) between the LPE and APE groups (Table 1).

**Table 1:** Comparisons of clinical parameters between LPE group and APE groups

Variables	LPE (N = 252)	APE (N = 663)	*P* value
Age (years)	30 (20–51)	30 (20–59)	0.076
≤25	56 (22.2)	86 (13.0)	
26–30	78 (31.0)	294 (37.6)	
>30	118 (46.8)	328 (49.5)	
IIEF−5			0.653
≥22	21 (8.3)	45 (6.8)	
12–21	173 (68.7)	474 (71.5)	
8–11	42 (16.7)	112 (16.9)	
1–7	16 (6.3)	32 (4.8)	
PEDT			<0.001
≤8	29 (11.5)	139 (21.0)	
9–10	85 (33.7)	281 (42.4)	
≥11	138 (54.8)	243 (36.7)	
IELT (min)			<0.001
IELT<1	205 (81.3)	259 (39.1)	
1≤IELT<2	28 (11.1)	217 (32.7)	
2≤IELT<3	19 (7.5)	187 (28.2)	
Masturbation			0.091
No/Unknown	55(21.8)	181 (27.3)	
Yes	197(78.2)	482 (72.7)	
FSD			0.039
≤50%	123(48.8)	374 (56.4)	
>50%	129(51.2)	289 (43.6)	
PHQ−9			0.513
0–4	67 (26.6)	202 (30.5)	
5–9	91 (36.1)	243 (36.7)	
10–14	60 (23.8)	132 (19.9)	
15–19	26 (10.3)	58 (8.7)	
20–27	8 (3.2)	28 (4.2)	
GAD−7			0.495
0–4	121 (48.0)	338 (51.0)	
5–9	97 (38.5)	227 (34.2)	
10–14	25 (9.9)	63 (9.5)	
15–21	9 (3.6)	35 (5.3)	
EPQ‐RSC(P)			<0.001
40–60	208 (82.5)	345 (52.0)	
<40	15 (6.0)	169 (25.5)	
>60	29 (11.5)	149 (22.5)	
EPQ‐RSC(E)			0.731
40–60	39 (15.5)	113 (17.0)	
<40	165 (65.5)	436 (65.8)	
>60	48 (19.0)	114 (17.2)	
EPQ‐RSC(N)			0.379
40–60	52 (20.6)	132 (19.9)	
<40	161 (63.9)	402 (60.6)	
>60	39 (15.5)	129 (19.5)	
EPQ‐RSC(L)			0.166
40–60	153 (60.7)	434 (65.5)	
<40	51 (20.2)	100 (15.1)	
>60	48 (19.0)	129 (19.5)	

Abbreviations: EPQ‐RSC(E), EPQ‐RSC(extraversion), EPQ‐RSC(N) EPQ‐RSC(neuroticism); EPQ‐RSC(L), EPQ‐RSC(lie). EPQ‐RSC(P), Eysenck Personality Questionnaire‐Revised Short Scale for Chinese(psychoticism); FSD, Frequency of sexual desire; GAD‐7, Generalized Anxiety Disorder‐7; IELT, Intra‐vaginal Ejaculation Latency Time; IIEF‐5, 5‐item International Index Erectile Function; PEDT, Premature Ejaculation Diagnostic Tool; PHQ‐9, Patient Health Questionnaire‐9.

### 3.2 Independent predictors for APE

Age, PEDT, IELT, FSD, and EPQ‐RSC(P) were significantly associated with APE (*P* < 0.05) according to the univariate analysis. PEDT was excluded in the multivariate analysis due to significant negative correlation shown between PEDT and IELT (r = −0.362, *P* < 0.001) in the Spearman correlation analysis. Results of the multivariate analysis showed that age, IELT, FSD, and EPQ‐RSC(P) were all independent predictors for APE in PE patients (Table 2). Characteristics of independent predictors between the LPE and APE group were shown in Table 3.

**Table 2:** Univariate and multivariate logistic regression analyses of variables

Variables	Univariate analysis		Multivariate analysis	
	OR (95% CI)	*P* value	OR (95% CI)	*P* value
Age (years)		0.002		0.011
≤25	1 (Ref)		1 (Ref)	
26–30	2.079 (1.363–3.170)	0.001	2.086 (1.281–3.399)	0.003
>30	1.810 (1.217–2.692)	0.003	1.773 (1.114–2.822)	0.016
IIEF−5		0.655		
≥22	1 (Ref)			
12–21	1.279 (0.740–2.208)	0.378		
8–11	1.244 (0.664–2.331)	0.495		
1–7	0.933 (0.422–2.062)	0.865		
PEDT		<0.001		
≤8	1 (Ref)			
9–10	0.690 (0.432–1.101)	0.120		
≥11	0.367 (0.234–0.577)	<0.001		
IELT (min)		<0.001		<0.001
IELT<1	1 (Ref)		1 (Ref)	
1≤IELT<2	6.134 (3.974–9.469)	<0.001	6.563 (4.172–10.324)	<0.001
2≤IELT<3	7.790 (4.695–12.924)	<0.001	6.942 (4.107–11.734)	<0.001
Masturbation				
No/Unknown	1 (Ref)			
Yes	0.743 (0.527–1.049)	0.092		
FSD				
≤50%	1 (Ref)		1 (Ref)	
>50%	0.903 (0.820–0.995)	0.040	0.625 (0.446–0.876)	0.006
PHQ−9		0.515		
0–4	1 (Ref)			
5–9	0.886 (0.614–1.278)	0.516		
10–14	0.730 (0.483–1.101)	0.134		
15–19	0.740 (0.432–1.268)	0.273		
20–27	1.161 (0.505–2.670)	0.726		
GAD−7		0.498		
0–4	1 (Ref)			
5–9	0.838 (0.611–1.149)	0.272		
10–14	0.902 (0.543–1.499)	0.691		
15–21	1.392 (0.650–2.981)	0.394		
EPQ‐RSC(P)		<0.001		<0.001
40–60	1 (Ref)		1 (Ref)	
<40	6.793 (3.898–11.837)	<0.001	5.908 (3.298–10.585)	<0.001
>60	3.098 (2.008–4.778)	<0.001	3.162 (1.989–5.027)	<0.001
EPQ‐RSC(E)		0.731		
40–60	1 (Ref)			
<40	0.912 (0.608–1.368)	0.656		
>60	0.820 (0.499–1.346)	0.432		
EPQ‐RSC(N)		0.381		
40–60	1 (Ref)			
<40	0.984 (0.680–1.423)	0.930		
>60	1.303 (0.806–2.108)	0.281		
EPQ‐RSC(L)		0.168		
40–60	1 (Ref)			
<40	0.691 (0.471–1.015)	0.060		
>60	0.947 (0.648–1.384)	0.780		

Abbreviations: CI, confidence interval; EPQ‐RSC(E), EPQ‐RSC(extraversion); EPQ‐RSC(L), EPQ‐RSC(lie); EPQ‐RSC(N), EPQ‐RSC(neuroticism); EPQ‐RSC(P), Eysenck Personality Questionnaire‐Revised Short Scale for Chinese(psychoticism); FSD, Frequency of sexual desire; GAD‐7, Generalized Anxiety Disorder‐7; IELT, Intra‐vaginal Ejaculation Latency Time; IIEF‐5, 5‐item International Index Erectile Function;; OR, odd ratio; PEDT, Premature Ejaculation Diagnostic Tool; PHQ‐9, Patient Health Questionnaire‐9; Ref, reference.

**Table 3:** Characteristics of independent predictors between the LPE and APE group

Variables	LPE group(N = 252)	APE group(N = 663)	*P* value
Age(years)	31.00 ± 6.51	31.67 ± 5.98	0.035
EPQ‐RSC(P)	2 (1–3.75)	2 (1–3)	0.971
FSD	4 (3–4)	3 (3–4)	0.024
IELT (min)			<0.001
IELT<1	259 (39.1)	259 (39.1)	
1≤IELT<2	217 (32.7)	217 (32.7)	
2≤IELT<3	187 (28.2)	187 (28.2)	

Normally distributed variable (Age) was expressed as the mean ±SD and analyzed by Student's t test, while nonnormally distributed variables [EPQ‐RSC(P) and FSD)] were expressed as the median (interquartile range) and analyzed by Mann–Whitney U tests. Categorical data (IELT) were presented as frequencies (proportions) and were compared between subgroups by the Chi‐squared test. We used raw scores of EPQ‐RSC(P) for analysis.

Abbreviations: EPQ‐RSC(P), Eysenck Personality Questionnaire‐Revised Short Scale for Chinese(psychoticism); FSD, Frequency of sexual desire; IELT, Intra‐vaginal Ejaculation Latency Time.

### 3.3 Construction and evaluation of the nomogram

**Figure 1:**
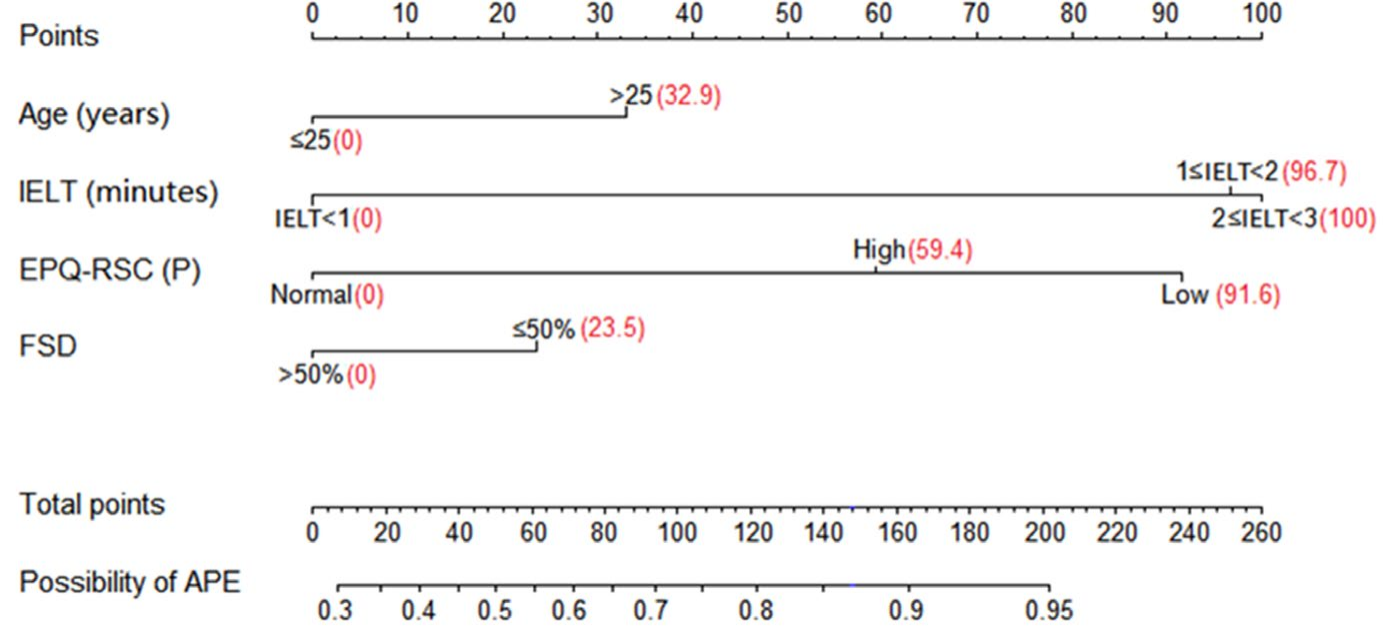
Nomogram for predicting the possibility of APE in PE patients. IELT Intra‐vaginal Ejaculation Latency Time EPQ‐RSC(P) Eysenck Personality Questionnaire‐Revised Short Scale for Chinese(psychoticism) FSD Frequency of sexual desire APE acquired premature ejaculation

**Figure 2:**
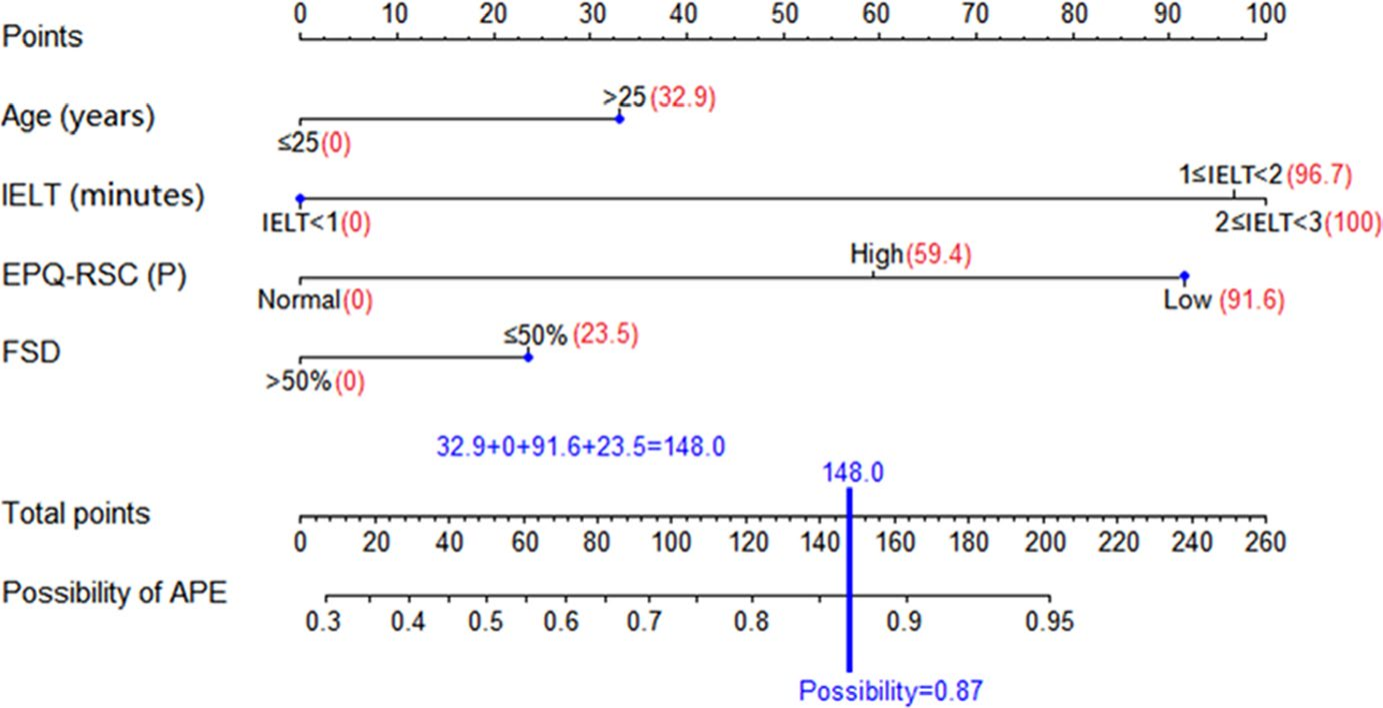
Example estimation of the possibility of being APE in a PE patient whose age>25, IELT<1, EPQ‐RSC(P) score was Low, and FSD ≤50%, his total points were calculated to be 148.0 (32.9 + 0 +91.6 + 23.5 = 148.0). Then, his possibility of being APE in PE patients was 87%. IELT Intra‐vaginal Ejaculation Latency Time EPQ‐RSC(P) Eysenck Personality Questionnaire‐Revised Short Scale for Chinese(psychoticism) FSD Frequency of sexual desire APE acquired premature ejaculation

The nomogram was constructed by integrating all independent predictors for APE according to the results of the multivariate logistic regression analysis. IELT and EPQ‐RSC(P) exhibited dominant influences, while age and FSD exhibited moderate impacts on the predictive outcome as shown in Figure 1. The usage of our nomogram is expressed by the following example (Figure 2). If the four variables of an PE patient were 1) Age>25 years, 2)IELT<1 minute, 3)EPQ‐RSC(P) score of Low, and 4)FSD≤50%, his total points were calculated to be 148.0 (32.9 + 0 +91.6 + 23.5 = 148.0), which indicates his possibility of being APE in PE patients is about 87%.

**Figure 3:**
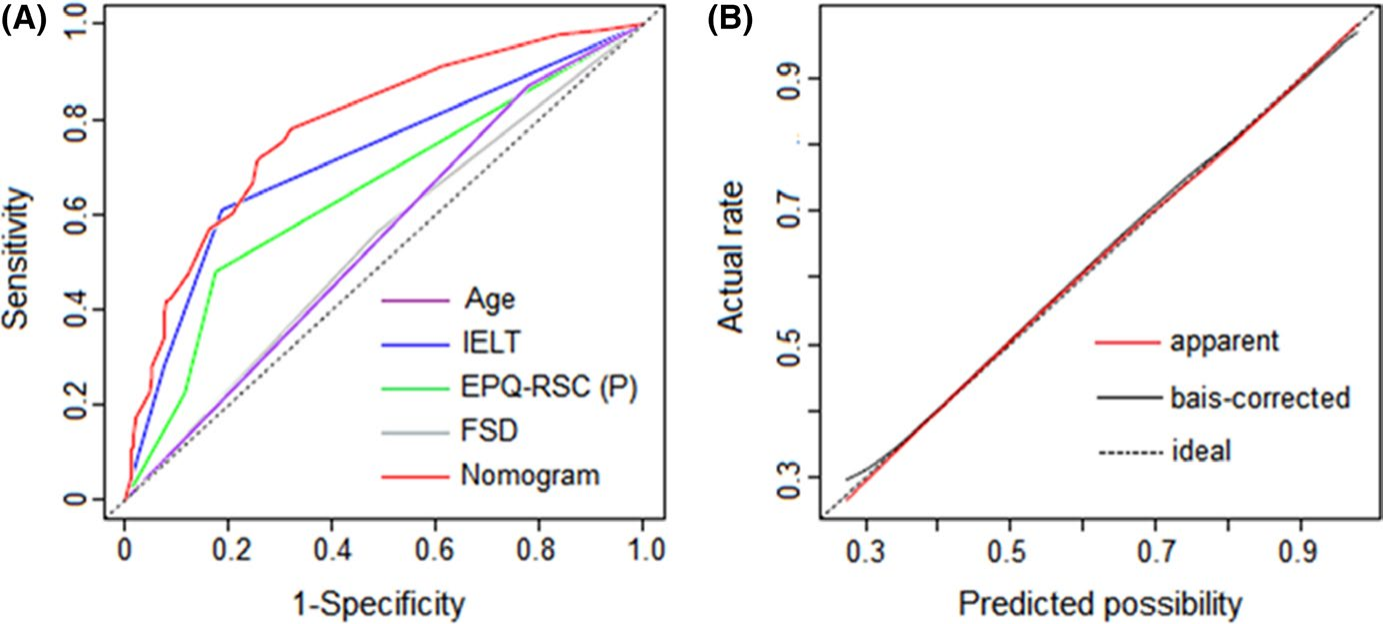
(A) ROC curves of the nomogram and its constituted variables. (B) Calibration plot of the nomogram. The dotted line represents the performance of an ideal nomogram (predicted possibility perfectly corresponds to the actual rate). The red solid line indicates the apparent accuracy of the nomogram without correction for overfitting. The black solid line is the bootstrap‐corrected nomogram. ROC receiver operating characteristic IELT Intra‐vaginal Ejaculation Latency Time EPQ‐RSC(P) Eysenck Personality Questionnaire‐Revised Short Scale for Chinese(psychoticism) FSD Frequency of sexual desire

Table 4 and Figure 3A show that the accuracy of our nomogram was 0.782 (95% CI: 0.723–0.841), which was higher than models established by integrating any 1, 2, or 3 of the variables among age, IELT, FSD, and EPQ‐RSC(P). Moreover, we achieved an excellent agreement between nomogram prediction and the actual situation in the calibration plot (Figure 3B).

**Table 4:** Discriminatory ability of predictive models

Constituted variables	AUC	Constituted variables	AUC
Age	0.546	IELT+EPQ‐RSC(P)	0.761
IELT	0.715	FSD+EPQ‐RSC(P)	0.659
FSD	0.538	Age+IELT+FSD	0.751
EPQ‐RSC(P)	0.645	Age+IELT+EPQ‐RSC(P)	0.773
Age+IELT	0.735	Age+FSD+EPQ‐RSC(P)	0.676
Age+FSD	0.573	IELT+FSD+EPQ‐RSC(P)	0.774
Age+EPQ‐RSC(P)	0.668	Age+IELT+FSD+EPQ‐RSC(P)	0.782
IELT+FSD	0.736		

Abbreviations: AUC, area under the receiver operating characteristic curve; IELT, Intra‐vaginal Ejaculation Latency Time, FSD Frequency of sexual desire; EPQ‐RSC(P), Eysenck Personality Questionnaire‐Revised Short Scale for Chinese(psychoticism).

## 4 DISCUSSION

It is necessary to distinguish these PE subtypes in clinical practice, since treatments of APE and LPE are evidently different. Currently, the diagnosis and classification of PE are mainly based on medical and sexual histories of patients, where IELT plays a dominant role. However, this approach may have relatively lower accuracy than a nomogram. To date, a diagnostic nomogram capable of intuitively and accurately predicting the risk of APE in PE patients has not yet been established, to the best of our knowledge. Furthermore, independent predictors of APE have not been elucidated completely.

The IELT of LPE patients was mainly concentrated in the <1 min group (81.3%), while 60.9% of APE patients were in the 1–3 min group in this study. This was consistent with ISSM’s definition of APE and LPE,^4^ which considered 1 minute and 3 minutes as the cutoff points, respectively. However, Côté‐Léger et al.^25^ reported that there was no significant difference in self‐rated IELT between patients with APE and LPE (*P* = 0.72). The differences may be explained by the fact that their results were based on an online survey rather than an in‐depth, face‐to‐face interview, and because the Mann–Whitney U test was performed on a small sample size. Of note, in our study PEDT showed a significant negative correlation with IELT (r = −0.362, *P* < 0.001), which was highly consistent with that performed by Huang et al.^19^ (r = −0.396, *P* < 0.001). It was excluded from the multivariate analysis for this reason.

A Chinese study by Gao et al.^26^ reported that APE patients were of a statistical higher mean age than were LPE patients (38.15 ± 9.21 years *vs*. 45.72 ± 12.82 years, *P* < 0.001). Age >25 years was further identified as an independent risk predictor for APE in our large‐sample‐based study. Such association suggests that early age (puberty or adolescence) of LPE may be attributed to neurobiology and genetics.^27^, ^28^, ^29^

Interestingly, patients with normal‐EPQ‐RSC(P) scores were more likely to be LPE patients, while those with abnormal scores, especially those which are low, were more likely to be APE patients. Compared to LPE patients, APE patients appeared to be less concentrated in the general score of PE patients along the EPQ‐RSC(P) scale. Our study is the first to report such findings. Two attributing factors have been suggested; 1) Psychoticism is the most heterogeneous of the personality dimensions proposed by Eysenck.^30^ LPE occurs earlier than APE and is not easy to be affected by acquired factors, and is mainly related to genetic and neurobiological factors. LPE should hence be relatively stable and resistant to change. 2) APE is mainly secondary to interventions for conditions which may have substantial impacts on the stability of personality, such as psychological or urinary disorders.

Testosterone is a clear determinant for sexual motivation, whose level is closely related to FSD.^31^ The fact that testosterone levels in patients with APE are significantly lower than those in patients with LPE (3.34 ± 1.56 vs. 4.47 ± 1.60 ng/mL) was confirmed by Tahtali İN,^32^ and it may contribute to the low FSD observed in APE compared with LPE. Furthermore, low testosterone level is associated with an overall tendency toward longer ejaculation,^33^ which also explains the longer IELT observed in APE patients than in LPE patients. Another study of Zacharie N^34^ confirmed that there was a significant reduction (*P* < 0.001) in Ejaculatory Latency (EL) in hypogonadal adult male rats treated with testosterone enanthate(3.6 mg/kg) for 14 days, which validated the negative correlation between testosterone levels and ejaculation latency in animal study.

Nomograms, graphical representations of multivariable models, have been proven to be effective in predicting the individual chance of experiencing certain events, such as erectile function recovery after radical prostatectomy,^35^ improvement of clinical global impression in LPE Patients Treated With Dapoxetine,^12^ and natural conception at various time points among couples diagnosed with unexplained subfertility.^36^ No nomogram has been established to predict the possibility of APE in patients with PE to our knowledge. The nomogram established in this study, combined with the patient's medical history, will be more accurate for distinguishing the 2 types of PE than any single factor included in the nomogram combined with the medical history. Obtaining a cure for PE was the primary goal for all 915 patients, which ensured that the data provided were true and reliable. Our nomogram has proven to be a relatively objective tool for the classification of PE and, hence, it offers a promising role as a reference for the treatment and prognosis of patients.

However, this study still had several limitations. The stop‐watch method was not used for measurement of IELT in this study due to inconvenience. Instead, in‐depth interviews with each patient were conducted to ensure that the medical history and self‐reported IELT were relatively accurate. Furthermore, the universal applicability our nomogram may have been affected to some extent because it was not externally validated. Nonetheless, the sample size of this study was large, and included patients from regions across the country, which provided a good representation of the PE population in China.

## 5 CONCLUSION

This large‐sample study identified 4 independent predictors for APE, and demonstrated that there are potential significant differences in psychoticism between LPE and APE patients for the first time. Furthermore, this is the first established predictive nomogram for APE in PE patients with good discrimination and calibration. Although more studies are required to verify its universal applicability in clinical practice, our nomogram is promising and could be clinically valuable.

## CONFLICTS OF INTEREST

None of the authors have any conflicts of interest to disclose.

## AUTHORS CONTRIBUTIONS

Conception and design: JLY, and MG. Data acquisition: LZ, XLD, and MG. Data analysis and interpretation: LZ, GDH, and XLD. Manuscript drafting: LZ, and GDH. Critical revision of the manuscript: DEJ, PM, JRY, and TJ. Statistical analysis: XLD, GDH and LZ. Figure design: GDH. Supervision: FL.
